# Attentional Control in Subclinical Anxiety and Depression: Depression Symptoms Are Associated With Deficits in Target Facilitation, Not Distractor Inhibition

**DOI:** 10.3389/fpsyg.2020.01660

**Published:** 2020-07-22

**Authors:** Alexandra C. Pike, Frida A. B. Printzlau, Alexander H. von Lautz, Catherine J. Harmer, Mark G. Stokes, MaryAnn P. Noonan

**Affiliations:** ^1^Department of Psychiatry, University of Oxford, Oxford, United Kingdom; ^2^Institute of Cognitive Neuroscience, University College London, London, United Kingdom; ^3^Department of Experimental Psychology, University of Oxford, Oxford, United Kingdom; ^4^Bernstein Center for Computational Neuroscience Berlin, Berlin, Germany; ^5^Oxford Health National Health Service Foundation Trust, Warneford Hospital, Oxford, United Kingdom

**Keywords:** attention, anxiety, depression, attentional bias, target facilitation

## Abstract

Mood and anxiety disorders are associated with deficits in attentional control involving emotive and non-emotive stimuli. Current theories focus on impaired attentional inhibition of distracting stimuli in producing these deficits. However, standard attention tasks struggle to separate distractor inhibition from target facilitation. Here, we investigate whether distractor inhibition underlies these deficits using neutral stimuli in a behavioral task specifically designed to tease apart these two attentional processes. Healthy participants performed a four-location Posner cueing paradigm and completed self-report questionnaires measuring depressive symptoms and trait anxiety. Using regression analyses, we found no relationship between distractor inhibition and mood symptoms or trait anxiety. However, we find a relationship between target facilitation and depression. Specifically, higher depressive symptoms were associated with reduced target facilitation in a task-version in which the target location repeated over a block of trials. We suggest this may relate to findings previously linking depression with deficits in predictive coding in clinical populations.

## Introduction

Attention is a core cognitive mechanism for optimizing information processing and failures of attentional control over emotive as well as non-emotive stimuli are consistently found in mood and anxiety disorders (Bar-Haim et al., 2007; Eysenck et al., 2007; McDermott and Ebmeier, 2009; Marazziti et al., 2010; Peckham et al., 2010; Shi et al., 2019). Failures of attention can be attributed to several component mechanisms (e.g., facilitation of goal-relevant information and/or inhibition of distractors) and disentangling their relative contribution to failures of attention in mood and anxiety disorders may be critical to understanding the etiology and developing effective treatments (Clarke et al., 2013). Here, we use a novel cognitive paradigm with mood-neutral stimuli to quantify two dissociable attentional mechanisms: target facilitation and distractor inhibition (Noonan et al., 2016). Specifically, we test whether these two component mechanisms of general attention are related to mood-relevant traits: anxiety and depression.

Attentional bias (toward threat-related or negative information) is one of the key cognitive markers in both major depressive disorder and anxiety disorders (Bar-Haim et al., 2007; Peckham et al., 2010), and may contribute to the development and/or maintenance of these disorders (Van Bockstaele et al., 2014; Disner et al., 2017). Additionally, an increasing body of literature suggests attentional deficits in mood and anxiety disorders are not always specific to emotive or disorder-relevant stimuli (“hot cognition”), but may reflect more general impairments in “cold cognition” including attentional control (Fox, 1994; Eysenck et al., 2007; Bishop, 2009; McDermott and Ebmeier, 2009; Marazziti et al., 2010; Roiser and Sahakian, 2013).

However, attentional control is multi-faceted, with distinctions drawn between attentional engagement (initial attentional capture), disengagement (removing attention) and switching (altering the focus of attention between stimuli). Failures of attention in mood and anxiety disorders have often been interpreted as particularly related to difficulties in inhibiting distractors. For example, the classic Posner cueing paradigm has been used to disentangle component mechanisms of attentional biases in subclinically anxious and depressed individuals and suggest a selective impairment in disengaging from threat, as opposed to attentional engagement or switching (Yiend and Mathews, 2001; Koster et al., 2005; but cf. Koster et al., 2006). Similarly, studies investigating deficits in “cold cognition” have specifically linked anxiety and depression symptomatology to deficits in distractor inhibition or disengagement, using paradigms such as the antisaccade task (Derakshan et al., 2009; Ansari and Derakshan, 2011; De Lissnyder et al., 2011) negative priming (Fox, 1994; MacQueen et al., 2000) the flanker task (Bishop, 2009; Dillon et al., 2015), and visual search (Moran and Moser, 2015; Moran, 2016). While not necessarily always to be expected, in many cases the deficits in cold cognition mirror deficits observed under emotional contexts. For example, Moran and Moser (2015) showed behavioral slowing in trait-anxious participants in a visual search task using non-emotive distractor stimuli; echoing Rinck et al. (2003) who found individuals with higher trait-anxiety had slower search times to disorder-relevant distractor words.

However, increased distraction need not arise because of failures to directly suppress distractors themselves, but could also arise due to failures of the target to maintain its goal-relevant status. Standard attentional tasks, as those mentioned above, struggle to disentangle inhibitory distractor processing from facilitation of target processing (Leber et al., 2016; Noonan et al., 2016). Recently, Noonan et al. (2016) showed that distractor inhibition is not under the same flexible top-down control as target facilitation. In a four-location variant of the Posner cueing paradigm, we cued participants to the spatial position of a target, the spatial position of a distractor or gave no informative cue. While target cues resulted in faster target discrimination regardless of whether the target location varied flexibly on a trial-wise basis or was fixed for a block of trials, distractor cues were only effective when the distractor location was fixed and repeated over a block of trials. Furthermore, flexible target facilitation and distractor inhibition did not correlate between participants, suggesting they rely on distinct cognitive mechanisms. These findings may have implications for interpreting deficits of distractor inhibition during attentional disengagement in mood disorders, as it raises the alternative possibility that impairments are instead a failure to maintain and focus on current goals (target facilitation), particularly in the presence of competition (distractors).

In the present study, we use non-emotive stimuli within a validated four location Posner-cueing paradigm that differentiates facilitation and inhibition (Noonan et al., 2016) to identify relationships between “cold” attentional mechanisms and mood symptoms. In a large cohort of healthy volunteers, with varied scores on self-report depression and anxiety questionnaires, we tested the degree to which distractor inhibition, as distinct from target facilitation, predicted mood score. As there is no evidence that distractors can be suppressed through top-down control in this task (Noonan et al., 2016) here we focused explicitly on distractor inhibition induced by distractor location repetition (“Blocked” task), as well as target facilitation induced by both the flexible top-down cues (“Flexible” task), and the task in which the target location was fixed for the duration of a block.

We hypothesized a negative relationship between distractor inhibition and both depression symptom scores and trait anxiety scores, such that higher scores would relate to participants’ ability to inhibit their responses to distractors. We had no *a priori* hypotheses about the relationship between target facilitation and these mood symptom scores.

Our subclinical approach of testing individuals on the spectrum of mood disorders is validated by work indicating that anxiety and depression are not categorical disorders (i.e., either present or absent), but operate on a continuum (Helzer et al., 2006). One advantage of such an approach is that it can tease apart factors which precede a clinical diagnosis, and therefore potentially relate to symptom development and may offer treatment targets, and factors that maintain the “state” of being currently unwell (or indeed are due to side effects of the disorder or medication used to treat it). This approach has been validated in other cognitive domains such as value-guided learning (Browning et al., 2015).

To preview our results, we find no evidence that depressive scores or trait anxiety are associated with distractor inhibition. However, we do find evidence that impairments in target facilitation may be related to depressive symptoms.

## Materials and Methods

### Participants

Eighty healthy participants were recruited from Oxfordshire, United Kingdom, of which data is reported from 71 (41 female). The participants were the same as those described in Experiment 2 in Noonan et al. (2016). Nine participants were excluded in total. Two participants were excluded before analysis: one participant was unable to complete the tasks due to fatigue and another participant misunderstood the task instructions. As described in Experiment 2 (Noonan et al., 2016), seven participants made more than 20% errors on an embedded no-go sub task designed to encourage fixation. These participants were likely not consistently attending to the fixation cross and were therefore excluded from further analysis.

Information on previous prescription of medication to alter mood/emotions was obtained but was not used as an exclusion criterion. Participants would have been excluded if they were currently taking any medications (except contraceptives). None fulfilled this criterion. All participants had normal or corrected to normal eyesight. Data from the remaining 71 participants (41 female, 9 left handed, 2 ambidextrous) were included in the analysis.

All participants gave informed consent prior to taking part. The University of Oxford’s Central University Research Ethics Committee approved the experiment.

### Design, Stimulus, and Procedure

Prior to the main behavioral task participants completed (1) the trait questionnaire of the State Trait Anxiety Inventory (STAI-T; Spielberger, 1983), (2) the Beck Depression Inventory II (BDI-II; Beck et al., 1996), and (3) a questionnaire assessing factors associated with cognitive function and mental health: age, including level of education, family history of mental illness, history of prescribed psychotropic drugs, smoking and alcohol consumption. All questionnaires were completed securely online using Qualtrics software (Qualtrics, 2009).

The task and experimental apparatus have been described in Experiment 2 in Noonan et al. (2016) and are reiterated here. The attention tasks were performed on Dell laptops with a screen resolution of 1280 × 800 px, using MATLAB R2013b (Mathworks, 2013) or a Dell Optiplex 9020 PC projecting to a Samsung Sync-Master 2233 monitor (60 Hz refresh rate, 1680 × 1060 pixel) with MATLAB 2014a (Mathworks, 2014). MATLAB was supplemented by the Psychophysics Toolbox (Brainard, 1997). Pre-recorded verbal instructions were provided through individual headphones which participants could review if they were unsure about the task after 20 practice trials. Participants made target discrimination judgments, identifying a target either on its own or in the presence of a distractor. Targets were triangles or squares and distractors were overlaid triangles and squares. Targets and distractors could appear in any of four quadrants of the screen. Stimuli subtended 2.4 degrees of visual angle and were 7.3 degrees from the fixation cross. The inclusion of four possible stimulus locations disentangles target facilitation from distractor inhibition: in the standard 2-location task, a target location cue equally predicts the distractor location (and vice versa). Participants discriminated between the two target types through mouse button responses, as quickly and accurately as possible, and received auditory accuracy feedback.

In one version of the task the cued stimulus locations were flexible and varied on a trial-wise basis. On every trial of this flexible version of the task, the exogenous cue, a small white dot, was presented to one corner of the fixation cross. In another version of the task the cued stimulus was fixed for each block of trials. In this blocked version of the task, the cue appeared at the start of each block only, and, in the case of target or distractor cues, predicted the location of that stimulus throughout the block. Task order was counterbalanced between participants.

A total of 720 trials were grouped into 45 blocks of 16 trials. Participants were instructed to fixate on the cross in the center of the screen. In both tasks, the inter-trial interval (ITI) lasted 200, 400, or 600 ms after which the fixation cross changed color from black to white to signal the fixation period (1000 ms). At the beginning of that fixation window, in the flexible task only, the cue was presented for 100 ms. The collective ITI and fixation epochs were therefore equivalent across the two tasks. After the end of the fixation period, the stimuli would appear for 200 ms, but there was no time limit for responses. In both task versions participants were cued to the forthcoming location of the target (target cue conditions), the distractor (distractor cue conditions), or given no predictive cue (neutral cue conditions). On neutral cue trials in the flexible task, the cue was presented randomly and provided no predictive information for the forthcoming location of either stimulus. The blocked neutral cue condition was equivalent to the flexible neutral condition in that no predictive information was given. Finally, distractor presence was also manipulated in a block-wise manner. Distractor cues in distractor absent conditions indicate the location of an absent distractor and provides an estimate of the advantage in reducing target location uncertainty with distractor cueing.

**FIGURE 1:**
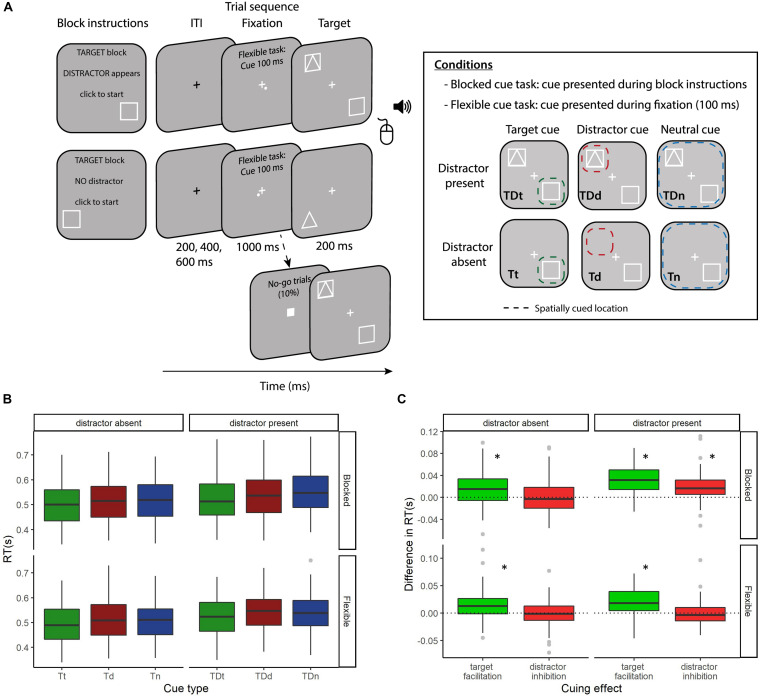
**(A)** Task schematic. Participants performed two versions of an adapted four-location Posner cuing task. In both task versions participants were instructed whether the forthcoming block would be a target, distractor or neutral cue condition. They were also informed whether a distractor would be present or not. In the blocked cueing task the participants were also informed of the location of the cued stimulus (target or distractor) which was valid for the block duration. Each trial began with a fixation cross (jittered: 200/400/600 ms), which would turn from black to white 1000 ms before stimulus onset. In the flexible cueing task a spatially predictive cue was presented in the corner of the white fixation cross for 100 ms. In neutral cue blocks the cue was presented randomly and did not provide predictive information. Targets were squares or triangles and distractors were superimposed squares and triangles. Participants responded whether the target was a triangle or square using the mouse buttons and accuracy feedback was provided with an auditory tone at the end of each trial. The three cue conditions and two distractor conditions resulted in six block types for each version of the task: (1) Target cuing condition, target present, no distractor (Tt), (2) Distractor cuing condition, target present, no distractor (Td), (3) Neutral cuing condition, target present, no distractor (Tn), (4) Target cuing condition, target and distractor present (TDt), (5) Distractor cuing condition, target and distractor present (TDd), and (6) Neutral cuing condition, target and distractor present (TDn). The dashed colored squares represent the spatially cued location (green = target, red = distractor, blue = neutral, for illustration purposes only) and illustrate how participants should optimally distribute their attention in the three conditions. The six conditions in each task would occur in randomized order an equal number of trials per block. **(B)** Mean RT across the six cue-types, faceted by task version (blocked or flexible) and by distractor presence (present or absent). **(C)** Cuing effects reported in Noonan et al. (2016) – target facilitation (the decrease in RT for target cuing vs. neutral cuing) and distractor inhibition (the decrease in RT for distractor location cuing vs. neutral cuing). These are again faceted by task version and distractor presence. Both **(B,C)** are boxplots of the difference in the median RTs of the cuing conditions of interest, displaying the median (central line), upper and lower quartiles (top and bottom hinges) and the largest and smallest values that are within 1.5*IQR of the upper and lower quartiles. Points beyond this are displayed as gray dots. ^∗^indicates significantly different from 0 at *p* < 0.05.

As shown in Figure 1, the three cue conditions and two distractor conditions resulted in six block types for each version of the task. Condition labels indicate which stimuli were present on any trial; target (T) or target + distractor (TD) and the cueing condition they were presented under; target (t), distractor (d), or neutral (n). The six conditions were (1) Target cuing condition, target present, no distractor (Tt), (2) Distractor cuing condition, target present, no distractor (Td), (3) Neutral cuing condition, target present, no distractor (Tn), (4) Target cuing condition, target and distractor present (TDt), (5) Distractor cuing condition, target and distractor present (TDd), and (6) Neutral cuing condition, target and distractor present (TDn). Conditions were randomized across the task. For analysis purposes, we focused on two *a priori* measures: distractor inhibition with the distractor present (TDn-TDd) and target facilitation with the distractor present (TDn-TDt).

On 10% of the trials a No-Go symbol was presented at fixation. 200 ms before stimulus onset, the fixation cross changed from a cross to a square, indicating that participants should not respond on the following trial. These trials were not analyzed but served as reassurance that subjects were consistently fixated. Participants were excluded if errors exceeded 20% on this sub-task (see above, seven participants were excluded). Participant accuracy was greater than 85% on the experimental tasks and no additional participants were excluded.

### Data Analyses

All analyses were performed in MATLAB (various versions) (Mathworks), and R (version 3.6.1). Data and scripts have been made available here: https://doi.org/10.17605/OSF.IO/XRDYB.

Outlier RTs were first removed using the Median Absolute Deviation method (Leys et al., 2013). The attentional cuing effects reported previously were analyzed with a series of paired *t*-tests between the median RTs of the cuing conditions of interest.

The relationships between variables measured by questionnaire were examined using Pearson’s Product Moment Correlations or Spearman’s rank-order correlations for continuous and non-continuous variables respectively.

Participant-level differences in the median RTs of the cuing conditions (TDn-TDd and TDn-TDt in the blocked condition and TDn-TDt in the flexible condition) were used as the dependent variable in a series of multiple regression analyses. Multiple regression analyses were then conducted using a stepwise procedure. A “null” model was constructed and included nuisance covariates: mean of median RTs across the tasks and conditions, age, gender, number of years of education completed, medication, family history of mental health problems, alcohol intake per week and cigarette use per week. The experimental model included the mean-centered STAI-T or BDI-II scores as well as the covariates from the null model. Comparison of model fits then examined the effects of the questionnaire scores controlling for nuisance variables.

In summarizing the effects we report the Δ*R*^2^ value reflecting the difference in *R*^2^ between the null model and the experimental model; the *F* statistic which represents the overall ability of the model to explain the data, and the corresponding *p*-value. In the cases where BDI-II or STAI-T are significant predictors of the outcome variable, we report the corresponding β weight, *t*- and *p*-value for that variable.

### Bayes Factor Analyses

We complement the classical frequentist statistics with Bayes Factor analyses. Bayesian statistics presented in the results were generated using the Bayesian equivalent of the frequentist test reported, using the BayesFactor package (version 0.9.12-4.2). The magnitude of the Bayes Factor can be used to interpret the strength of evidence: when 1 < BF < 3, the evidence is anecdotal, when 3 < BF < 10 the evidence is substantial, when 10 < BF < 30 the evidence is strong, when 30 < BF < 100 the evidence is very strong, and when BF > 100 the evidence is decisive (Jeffreys, 1961; Jarosz and Wiley, 2014).

These supporting analyses indicate the strength of evidence in favor of the null hypothesis H_0_ compared to H_1_ (reported here as *BF*_01_) and the strength of the evidence for the model of interest (reported as *BF*_10_).

## Results

### Demographics and Questionnaires

**FIGURE 2:**
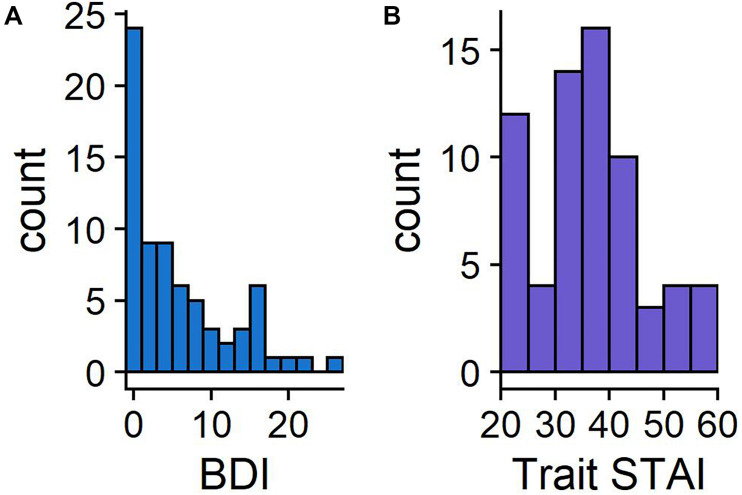
Histograms showing the frequency of BDI-II **(A)** and STAI-T scores **(B)**. BDI-II has a maximum of 63, with scores above 20 indicating moderate levels of depression. STAI-T scores can range between 20 and 80.

The distribution of BDI-II and STAI-T scores are presented in Figure 2, with the STAI-T scores comparable to the published norms (*M* = 38.2, *SD* = 11.1; Spielberger, 1983). BDI-II scores ranged from 0 to 27 (*M* = 6.2, *SD* = 6.6). All means and standard deviations of key metrics are reported in Table 1.

**TABLE 1:** Participant characteristics.

	**N**	**Mean**	***SD***	**Range**
BDI-II score	71	6.169	6.581	0–27
STAI-T score	71	38.169	11.121	22–62
Alcohol/week	71	8.887	9.678	0–40
Cigarettes/week	71*	6.296	20.005	0–100
Age	71	23.254	6.542	18–53
Education (years)	71	14.437	1.955	10–20

Mean and standard deviations of demographic and questionnaire variables. *58 participants did not smoke.

We examined relationships between demographic and questionnaire variables to identify overlapping constructs. In line with the known co-morbidity of anxiety and depression symptoms (Feldman, 1993) we report a significant positive correlation between BDI-II and STAI-T scores (*r*_69_ = 0.805, *p* < 0.001). Furthermore, we find that BDI-II score and alcohol intake are also correlated (*r*_69_ = 0.379, *p* = 0.001). These effects withstand Bonferroni correction (adjusted alpha level of 0.00139).

### Attention Task

Analysis of the impact of cue type and distractor presence on RT are reported in Noonan et al. (2016) [Experiment 2: repeated measures ANOVAs of distractor presence (present vs. absent) and cue contrast (target, distractor, neutral)]. For completeness, we describe the key results below and there is a summary in Figures 1B,C. As reported in Noonan et al. (2016), target facilitation effects were present in both task versions, with RTs faster when target location was cued compared to neutral cues (blocked distractor present: *t*_70_ = −10.80, *p* < 0.0001, *BF*_10_ = 4.3 × 10^13^; blocked distractor absent: *t*_70_ = −4.57, *p* < 0.0001, *BF*_10_ = 901.7, flexible distractor present: *t*_70_ = −6.35, *p* < 0.0001, *BF*_10_ = 6.5 × 10^5^; flexible distractor absent: *t*_70_ = −4.23, *p* < 0.0001, *BF*_10_ = 297). Distractor inhibition was only evident in the blocked task version. RTs were faster when the location of the distractor repeated over a block of trials compared to neutral trials (blocked distractor present: *t*_70_ = −5.96, *p* < 0.0001, *BF*_10_ = 1.5 × 10^5^). This effect was absent when the distractor was not presented (blocked distractor absent: *t*_70_ = −0.06, *p* = 0.952, *BF*_01_ = 7.65) and when the distractor cue varied trial-wise, indicating that distractor inhibition is not as flexible as top-down target facilitation (flexible distractor present: *t*_70_ = 0.25, *p* = 0.798, *BF*_01_ = 7.43; flexible distractor absent: *t*_70_ = 0.27, *p* = 0.784, *BF*_01_ = 7.39). Finally, blocked distractor inhibition and flexible target facilitation did not correlate between participants (*r*_69_ = 0.165, *p* = 0.168, *BF*_01_ = 1.53) suggesting these attentional cueing effects rely on different attentional control mechanisms (Noonan et al., 2016).

In the current experiment, we examined these attentional effects as a function of trait STAI-T scores and BDI-II scores.

### BDI-II and STAI-T Do Not Predict Distractor Inhibition, BDI-II Predicts Target Facilitation

Using multiple regression analyses, we examined the degree to which non-clinical trait anxiety and depression symptoms related to two key *a priori* cueing effects: distractor inhibition (TDn-TDt) in the blocked task, or target facilitation (TDn-TDt) in either flexible or blocked cueing task versions.

Contrary to our hypotheses, anxiety and depression scores were not predictive of distractor inhibition effects, with neither BDI-II nor STAI-T scores explaining a significant amount of task variance, no significant improvement over the null model by adding BDI-II or STAI-T, and the resulting models being non-significant in the blocked version [BDI: Δ*R*^2^ = 0.001, *R*^2^ = −0.11, *F*_(9,61)_ = 0.25, *p* = 0.98, *BF*_01_ = 1.81; STAI-T: Δ*R*^2^ = 0.003, *R*^2^ = −0.10, *F*_(9,61)_ = 0.27, *p* = 0.98, *BF*_01_ = 1.56].

**FIGURE 3:**
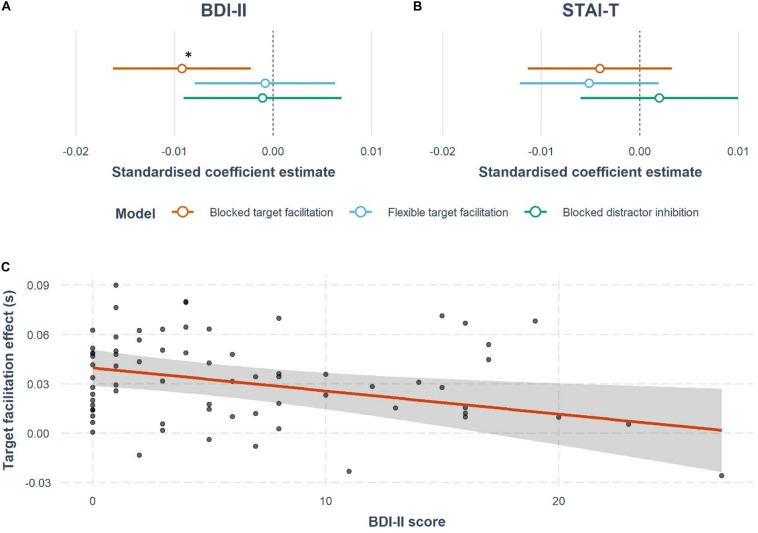
Results of the multiple regression analyses testing whether depression or anxiety affects target facilitation or distractor inhibition. Forest plots represent the estimated effects of **(A)** BDI-II scores and **(B)** STAI-T scores on cuing effects. These plots show the standardized coefficient estimates from the models discussed in the results section, including nuisance covariates of age, gender, cigarettes, and alcohol units per week, family history of mental health, and overall mean RT in all the tasks. There is a significant negative effect of BDI-II score on blocked target facilitation, marked with a *. **(C)** The regression slope for BDI-II and target facilitation in the blocked version of the task. Each point indicates a participant’s response, while the 95% confidence interval around the regression line are also shown.

By contrast, BDI-II scores predicted blocked target facilitation (Figures 3A,C) (β = −0.0014, *t* = −2.65, *p* = 0.01). The overall model was not significant [*R*^2^ = 0.04, *F*_(9,61)_ = 1.34, *p* = 0.23], but the model significantly improved compared to the null model with the addition of BDI-II score [Δ*R*^2^ = 0.096, Δ*F*_(1,61)_ = 7.03, *p* = 0.01, *BF*_10_ = 5.65]. Notably, the presence of a distractor is essential for this relationship (Tn-Tt *p*s > 0.5). However, STAI-T scores do not significantly predict target facilitation effects (Figure 3B), with no improvement seen from the null model by adding STAI-T score [STAI-T: Δ*R*^2^ = 0.02, *R*^2^ = −0.05, *F*_(9,61)_ = 0.65, *p* = 0.75, *BF*_01_ = 1.40]. Furthermore, neither BDI-II or STAI-T scores explained a significant amount of variance in target facilitation in the flexible version of the task [BDI: Δ*R*^2^ = 0.0007, *R*^2^ = 0.04, *F*_(9,61)_ = 1.32, *p* = 0.25, *BF*_01_ = 2.08; STAI-T: Δ*R*^2^ = 0.03, *R*^2^ = 0.07, *F*_(9,61)_ = 1.59, *p* = 0.14, *BF*_01_ = 1.03].

### No Relationship Between Overall RT and STAI-T/BDI-II Scores

Finally, to rule out a confounding relationship between general RT slowing and higher mood symptoms (Eysenck et al., 2007; Marazziti et al., 2010), we performed two multiple regression analyses on RTs across each condition, separately for each version of the task, covarying out demographic information (but not overall RT) as described above. Neither STAI-T nor BDI-II scores explained a significant amount of variance when added to a null model containing covariates in the blocked version of the task [BDI: Δ*R*^2^ = 0.006, *R*^2^ = 0.03, *F*_(8,62)_ = 1.29, *p* = 0.26, *BF*_01_ = 1.83; STAI-T: Δ*R*^2^ = 0.003, *R*^2^ = 0.03, *F*_(8,62)_ = 1.27, *p* = 0.28, *BF*_01_ = 1.50], or flexible version of the task [BDI: Δ*R*^2^ = 0.002, *R*^2^ = 0.006, *F*_(8,62)_ = 1.05, *p* = 0.41, *BF*_01_ = 1.88; STAI-T: Δ*R*^2^ = 0.0003, *R*^2^ = 0.004, *F*_(8,62)_ = 1.03, *p* = 0.42, *BF*_01_ = 2.12].

## Discussion

Target facilitation and distractor inhibition are governed by distinct neurocognitive mechanisms (Noonan et al., 2016). Here we explored the implications of this for understanding deficits of selective attention in mood disorders. Using an adapted non-emotive Posner cueing task that differentiates target facilitation and distractor inhibition in a subclinical population, we examined the degree to which individual differences in depression symptomology and trait anxiety were specifically associated with distractor inhibition. Contrary to our hypotheses, we found no evidence that subclinical depression scores or trait anxiety were associated with a reduced capacity to inhibit distractors. However, we did find evidence for a relationship between target facilitation and depressive symptoms, as measured by the BDI-II.

Specifically, in the blocked task-version, we showed individuals with higher depression symptomatology had reduced target facilitation effects. In this task targets are not explicitly cued but instead are presented repeatedly in the same (initially cued) location over the course of a block of trials. This may mean that additional mechanisms support optimal performance within the blocked task version compared to the flexible task. While the flexible task relies on working-memory based top-down control, performance in the blocked task may be supported by a combination of top-down control and more implicit mechanisms, such as predictive coding (Friston, 2010). While our task cannot fully orthogonalize effects of top-down and implicit mechanisms of target facilitation, the finding that individuals with high depressive symptomatology are less able to benefit from an increasingly predictable target location in the blocked task is consistent with past literature, linking depression to deficits in predictive coding (Chekroud, 2015; Barrett et al., 2016; Schutter, 2016; Badcock et al., 2017; Kube et al., 2020). For example, a deficit in updating an internal model in depressed individuals (Barrett et al., 2016; Schutter, 2016) may underlie the poorer use of predictable implicit target information as seen in the present task. Models which conceptualize depression in terms of active inference failures suggest that issues in learning from prediction errors result in negative emotional states (Joffily and Coricelli, 2013) although it remains to be tested whether deficits in predictive coding arise throughout perceptual processes in depression and may underlie the observed longer RTs in the current study when the target predictably repeated to the same location.

By contrast, we find no relationship between blocked target facilitation and anxiety. Past work has shown that subclinically anxious individuals have difficulty learning the causal statistics of the environment when making value-guided decisions in the context of changeable but predictable reward environments (Browning et al., 2015). The learning environment in that experiment used aversive stimuli and therefore the lack of effect in the present experiment, assuming similar statistical, expectation-based mechanisms partially support performance in the blocked task, may suggest the emotive nature of the stimuli is crucial to the manifestation of any effects. This interpretation would be further supported by literature showing deficits in attentional control for emotive, but not neutral stimuli, in anxious participants (Lichtenstein-Vidne et al., 2017). Of further note, Browning’s subclinical sample contained a similar distribution of STAI-T scores (*M* = 42.7, *SD* = 13.6, *Range* = 23–69) to the data presented here (*M* = 38.2, *SD* = 11.12, *Range* = 22–62) (Browning et al., 2015), providing reassurance that a null effect is unrelated to subclinical sample biases in anxiety scores. The distribution of our BDI-II scores are also comparable to published studies. For example, Koster and colleagues matched two groups of 15 subjects with BDI-II scores over 9 and 5 or under (Koster et al., 2005). More than 25% of our sample, which corresponds to 33 subjects, have BDI-II scores above the cut-off imposed by Koster to reflect relatively higher depressive symptomology.

More broadly, past studies that report general deficits in attentional control using neutral stimuli in anxiety (Fox, 1994; Eysenck et al., 2007; Bishop, 2009) and depression (McDermott and Ebmeier, 2009) often interpreted them as failures of inhibition. For example, negative priming (i.e., slowed responses to a location or item that was just ignored) is reduced in individuals with high trait anxiety (Fox, 1994) and depression (MacQueen et al., 2000). However, methodological challenges may limit the negative priming paradigm as a measure of inhibition, as the effect could also be related to feature and/or response conflict from previous trials (Macleod et al., 2003). Supporting this interpretation, we observe that the relationship between depression symptomology and reduced target facilitation was only present when distractors were competing for attentional resources. We note that other studies, using different measures of inhibitory control, such as the Attention Network Test, the Flanker task and the Stroop task in depression and anxiety (Pizzagalli et al., 2006; Holmes and Pizzagalli, 2008; Moriya and Tanno, 2009; Lichtenstein-Vidne et al., 2017) also do not find evidence for impaired distractor inhibition in anxiety and depression. However, an interesting point to consider is the degree to which compensatory neural mechanisms can be engaged by individuals with high levels of anxiety or depressive symptomatology to overcome potential behavioral deficits. Studies measuring neural responses during visual search with salient distractors in high and low anxious individuals have found some evidence in support of such a theory (Berggren and Derakshan, 2013; Gaspar and McDonald, 2018; but cf. Bishop, 2009; Ansari and Derakshan, 2011).

Our results may suggest a re-interpretation of “failures of inhibition” in depression as an impairment in the target maintaining its goal-relevant status in the presence of distractors, rather than distractor inhibition deficits *per se*. By using a paradigm that disentangles mechanisms of facilitation and inhibition in attentional control (Noonan et al., 2016) we are now able to demonstrate the clinical relevance of these distinct attentional mechanisms in understanding attentional control deficits in mood disorders. If distractor inhibition is not as flexible as top-down control and less reliant on working memory (Leber et al., 2016; Noonan et al., 2016), this will have implications for the cognitive models of anxiety and depression (e.g., Attentional Control Theory) that have assumed deficits in top-down, working-memory dependent, inhibitory control (Eysenck et al., 2007). Moreover, with cognitive treatments for depression, such as attentional bias modification with emotional stimuli, gaining traction in recent years (Mogoase et al., 2014), findings of general deficits in target facilitation through expectation, particularly in depression, may suggest treatment could focus on general goal-orientated information-processing (Cohen et al., 2016). If individuals with depression fail to maintain (or learn) the targets predictability in the presence of salient distractors then attentional bias modification training could explore treatments that promote implicit learning and attentional mechanisms.

One outstanding question that remains unanswered is the extent to which top-down distractor inhibition, should it exist, relates to mood disorder scores. One possibility is that if distractor inhibition is not as flexible as target enhancement then it needs more time to manifest than allowed in the current experimental design. However, Wang and Theeuwes (2018) recently found no evidence for flexible top-down distractor inhibition at long or short SOAs. Furthermore, longer SOA may aid re-orientating to uncued locations, as opposed to a direct distractor inhibition mechanism. In our review of alternative models of inhibition, we consider this re-orientating mechanism as a special case of facilitation (Noonan et al., 2018). Our task specifically included multiple uncued locations in order to dilute any benefit of re-orienting strategy. As discussed here we focus on a robust version of inhibition induced by increased expectations of distractor location and we find no evidence that increased measures of anxiety or depression are associated with deficits in distraction suppression. By contrast we report that target facilitation induced by increased expectations of target location correlated with depression scores. However, should flexible inhibition be observed via a different task or cueing paradigm then it would be important to examine its relationship to anxiety/depression scores.

## Conclusion

To conclude, we suggest that depressive symptomology is related to general deficits in attentional control. This deficit does not appear to be driven by distractor inhibition difficulties as we had expected, but by impairments in goal-directed information processing (target facilitation) in the presence of competing information. Experiments including mood-relevant stimuli could be conducted in the future, to examine whether the relationships we have found here might contribute to attentional biases toward negative or emotional stimuli. Our study highlights the importance in disentangling contributions of faciliatory and inhibitory information-processing mechanisms to better understand the etiology and maintenance of anxiety and depression disorders. The next step would involve further isolating top-down from implicit component mechanisms of target facilitation and elucidating their precise relationship with mood disorder symptomology.

## Data Availability Statement

All datasets generated for this study are available at https://doi.org/10.17605/OSF.IO/XRDYB.

## Ethics Statement

The studies involving human participants were reviewed and approved by the Oxford Central University Research Ethics Committee. All participants provided their written informed consent to participate in this study.

## Author Contributions

AP and FP as co-joint first authors designed, ran, analyzed, and wrote up the study. AL and CH were involved in the design and write up of the study. MS and MN were involved in the design, analysis, and write up of the study. All authors contributed to the article and approved the submitted version.

## Conflict of Interest

The authors declare that the research was conducted in the absence of any commercial or financial relationships that could be construed as a potential conflict of interest.
